# Supplementary material for: Peripheral sequestration of huntingtin delays neuronal death and depends on N-terminal ubiquitination
Source: Commun Biol. 2024 Aug 18;7:1014. doi: 10.1038/s42003-024-06733-1 (PMC11330980; doi:10.1038/s42003-024-06733-1)
Supplement: Supplementary file 2 — Supplemental Information [file 42003_2024_6733_MOESM2_ESM.pdf]

Peripheral sequestration of huntingtin delays neuronal death and depends on N-terminal ubiquitination

Ayub Boulos, Dunia Maroun, Aaron Ciechanover and Noam E. Ziv

Supplementary Figures 1 to 6

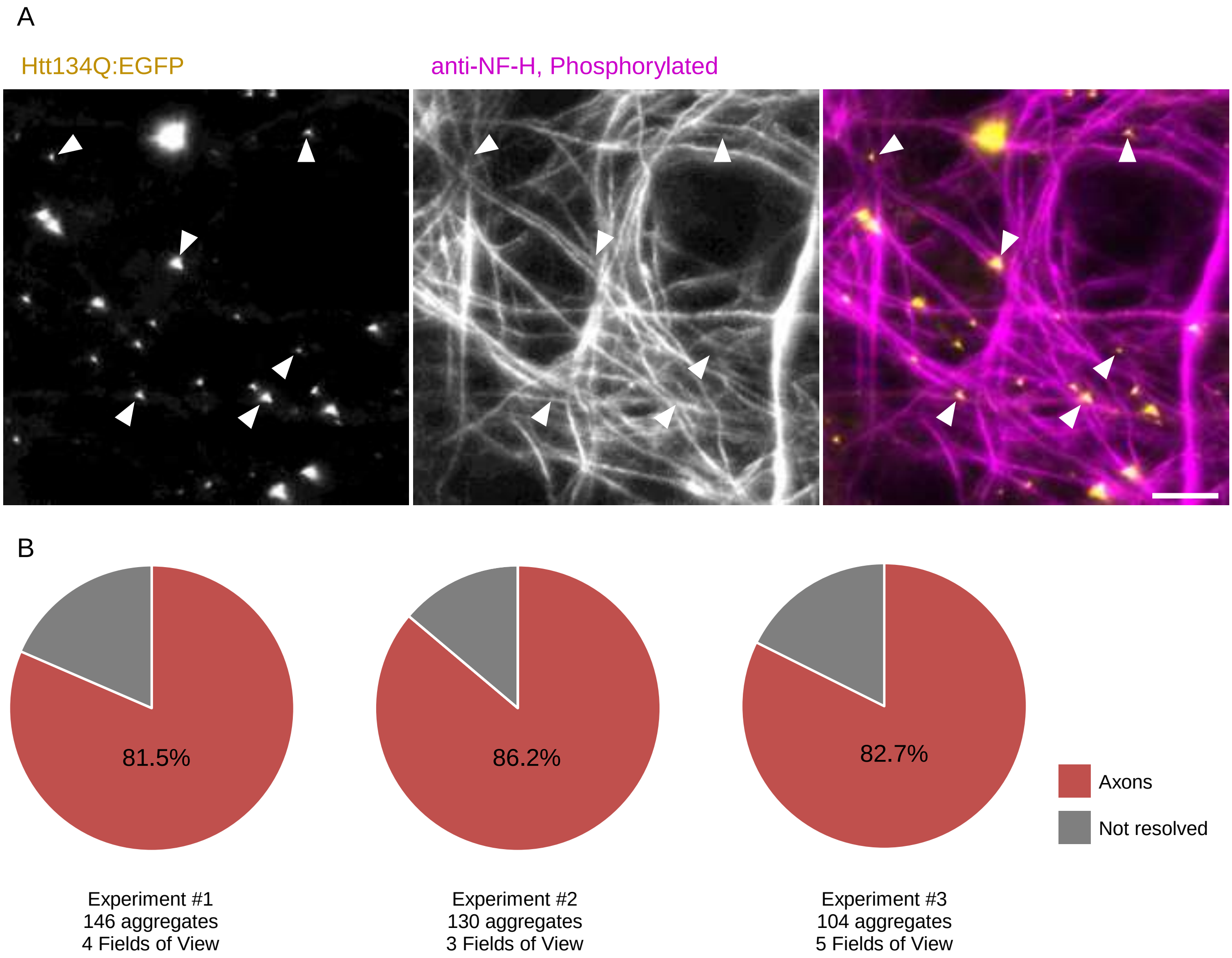

Supplementary Figure 1

The majority of Htt134Q:EGFP aggregates colocalize with axons. Cortical neurons plated on glass-bottom petri dishes expressing Htt134Q:EGFP were subjected to slow perfusion, a sterile stream of 5% CO_2 / 95% air mixture, and ~35-36°C for 2 days, after which they were fixed and stained with an antibody against phosphorylated Neurofilament heavy chain (NF-H) and imaged. A) A representative field of view showing numerous Htt134Q:EGFP aggregates (left panel) and anti NF-H labeling (middle panel). Arrowheads point to a number of Htt134Q:EGFP aggregates. **B)** Fraction of Htt134Q:EGFP aggregates that colocalized with NF-H positive neurites. Data from 380 aggregates from 12 fields of view from three experiments using neurons from two cell culture preparations. Scale bar: 10µm.

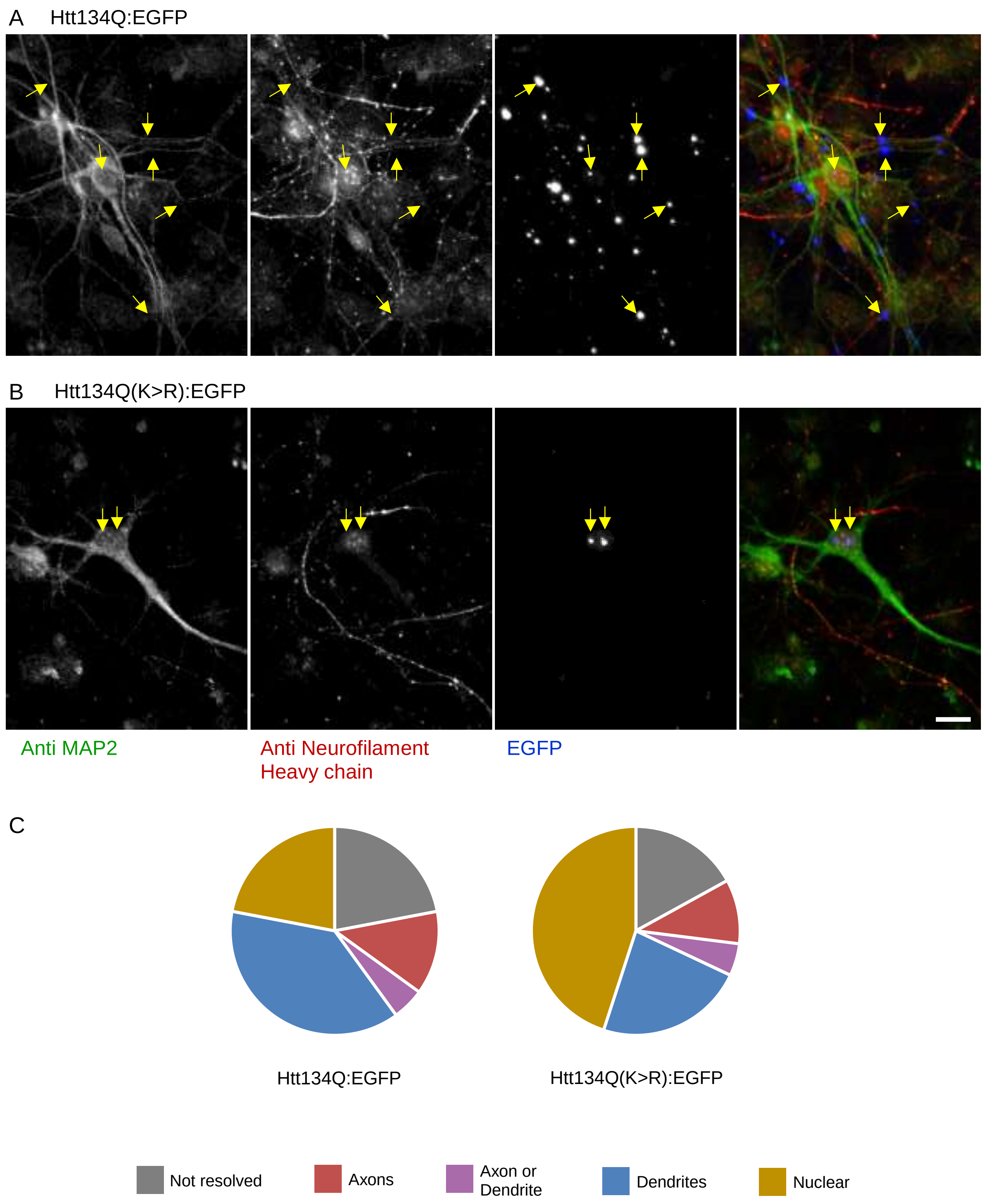

Supplementary Figure 2

Sub-cellular localization of mHtt aggregates in non-perfused neurons. Cortical neurons in culture were infected with either Htt134Q:EGFP or Htt134Q(K>R):EGFP lentiviral particles. 10 days post infection, cells were fixed stained using antibodies against Neurofilament Heavy chain and MAP2 (see Materials and Methods). **(A)** Representative images of neurons in preparations expressing Htt134Q:EGFP. **(B)** Representative images of neurons in preparations expressing Htt134Q(K>R):EGFP. **(C)** Aggregate distribution within neuronal compartments in preparations expressing Htt134Q:EGFP and Htt134Q(K>R):EGFP. Data from three independent experiments.

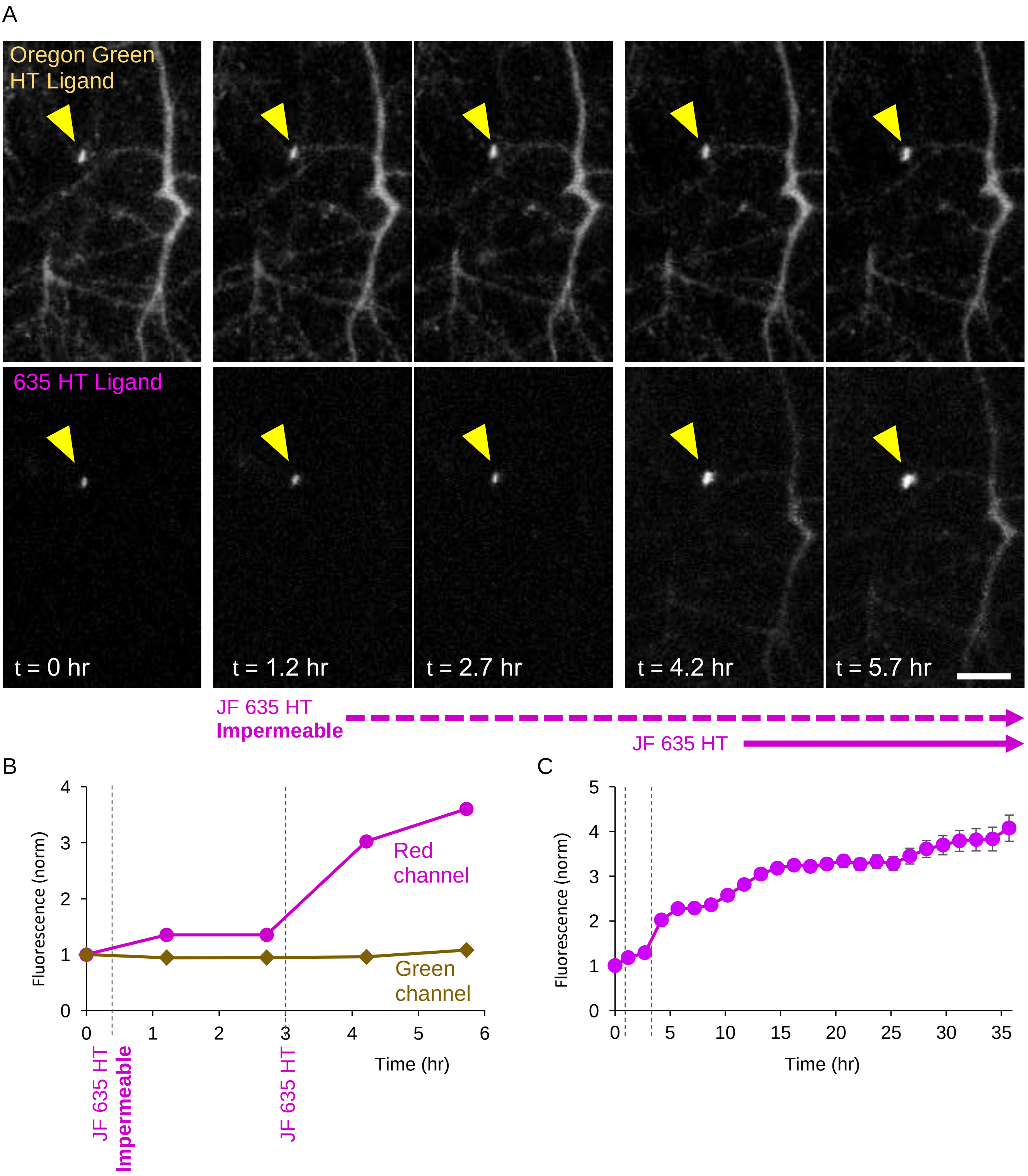

Supplementary Figure 3

mHtt aggregates are located inside cells. **(A)** Time-lapse images of cortical neurons expressing Htt134Q:HaloTag after labeling with a first HaloTag ligand (Oregon green HaloTag, top). A membrane impermeable ligand (JF635i-HT) was then added to the media. After about 3 hours, a third ligand (JF635-HT) was added as well. mHtt puncta indicated by the yellow arrow. Note that the initial fluorescence in the red (JF635i-HT/ JF635-HT) channel represents cross-talk arising from Oregon Green and is present before any red HaloTag ligands were added to the media. This cross talk was sometimes hard to avoid because of the extreme brightness of some aggregates. Scale bar: 20 µm. **(B)** Changes in the fluorescence intensities measured in the Oregon green and JF635i-HT/ JF635-HT channels for the aggregate indicated in A. Fluorescence values were normalized to values at t=0. **(C)** Average

changes in the fluorescence intensities in the JF635i-HT / JF635-HT channel for 82 aggregates from three independent experiments. Fluorescence values normalized to values at t=0. Vertical dashed lines indicate the times at which JF635i-HT (left) and JF635-HT (right) were added to the media. Bars, SEM.

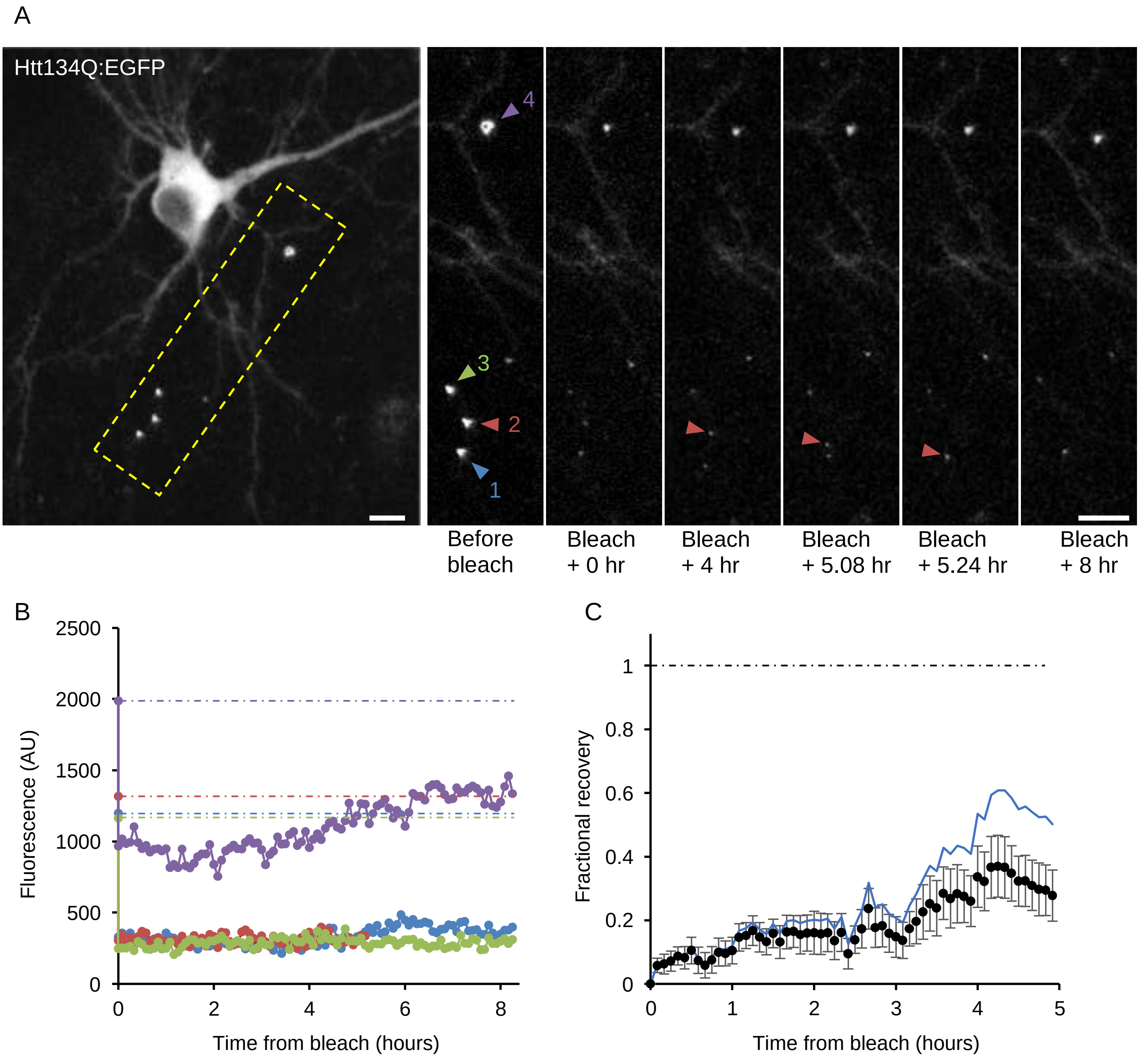

Supplementary Figure 4

Aggregate-associated Htt134Q:EGFP exchange measured by Fluorescence Recovery after Photobleaching (FRAP). (A) A neuron expressing mHtt:134Q (left-hand panel). Four bright aggregates are visible in this field of view. The right-hand panels show these aggregates at a higher magnification (region enclosed in dashed line on the left) before and after individually photobleaching these aggregates. Arrows indicate aggregates whose fluorescence is quantified in B. Note the migration and merging of aggregates #1 and #2. Scale bar: 10 µm. The dark spot in the center of aggregate 4 is an artifact of saturation. **(B)** Changes in EGFP fluorescence intensities for the four aggregates shown in A. **(C)** Average changes in EGFP fluorescence intensities of within photobleached aggregates from three independent experiments (37 aggregates). Fluorescence values normalized to values obtained before and immediately after photobleaching. Blue line indicates recovery curve after correcting for ongoing photobleaching associated with imaging during the recovery phase. Bars, SEM.

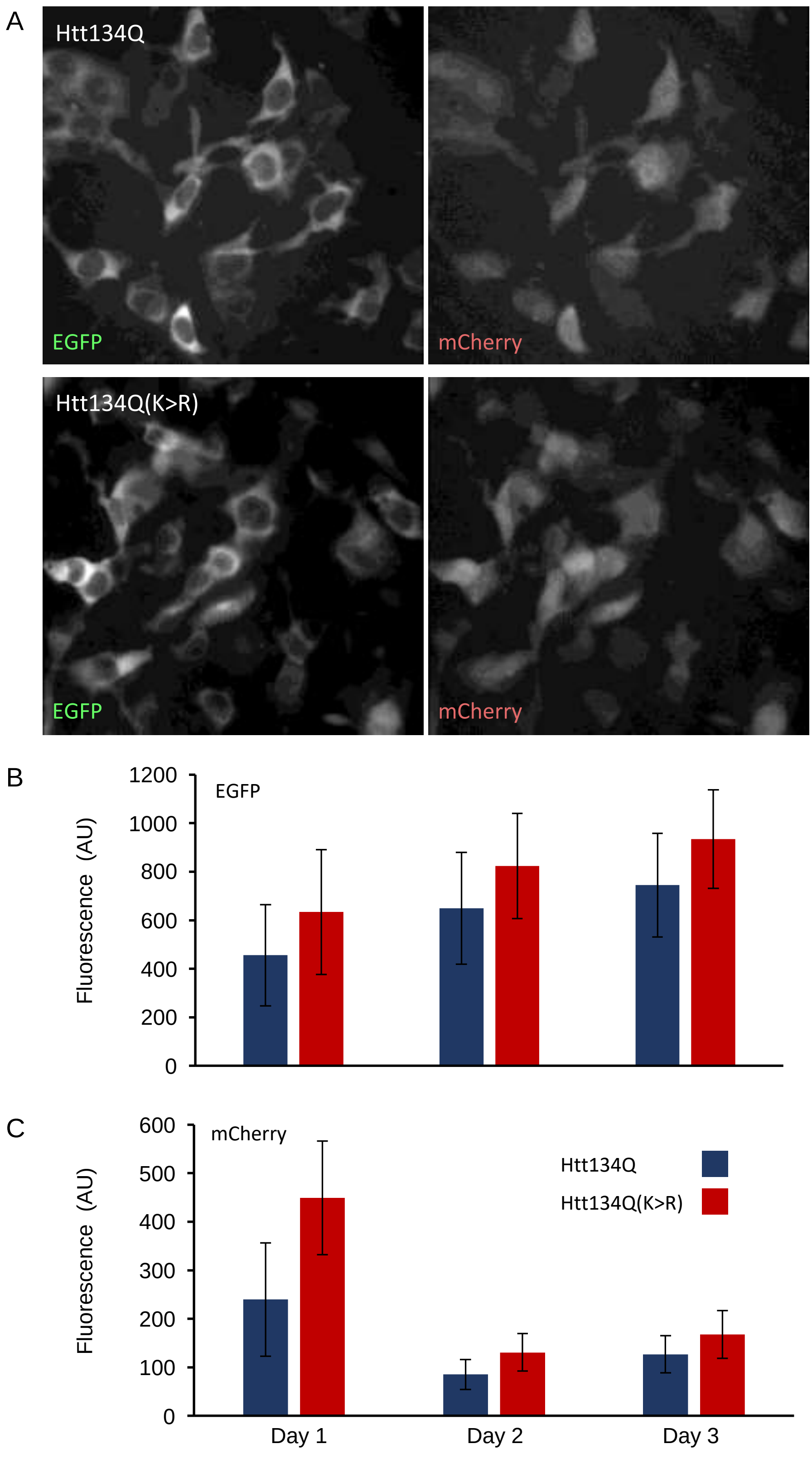

Supplementary Figure 5

Expression levels of Htt134Q:EGFP:T2A:mCherry and Htt134Q(K>R):EGFP:T2A:mCherry in HEK293 cells. HEK293 cells were infected with equal volumes of viral particles encoding for either Htt134Q:EGFP:T2A:mCherry or Htt134Q(K>R):EGFP:T2A:mCherry and imaging was carried out 1, 2 or 3 days post infection. **(A)** representative image of HEK293 cells expressing both constructs. **(B)** Fluorescence of EGFP and **(C)** mCherry for both viral vectors on each day. 5 fields of view for each day and condition. 3 separate dishes (one for each day). Bars, standard deviation.

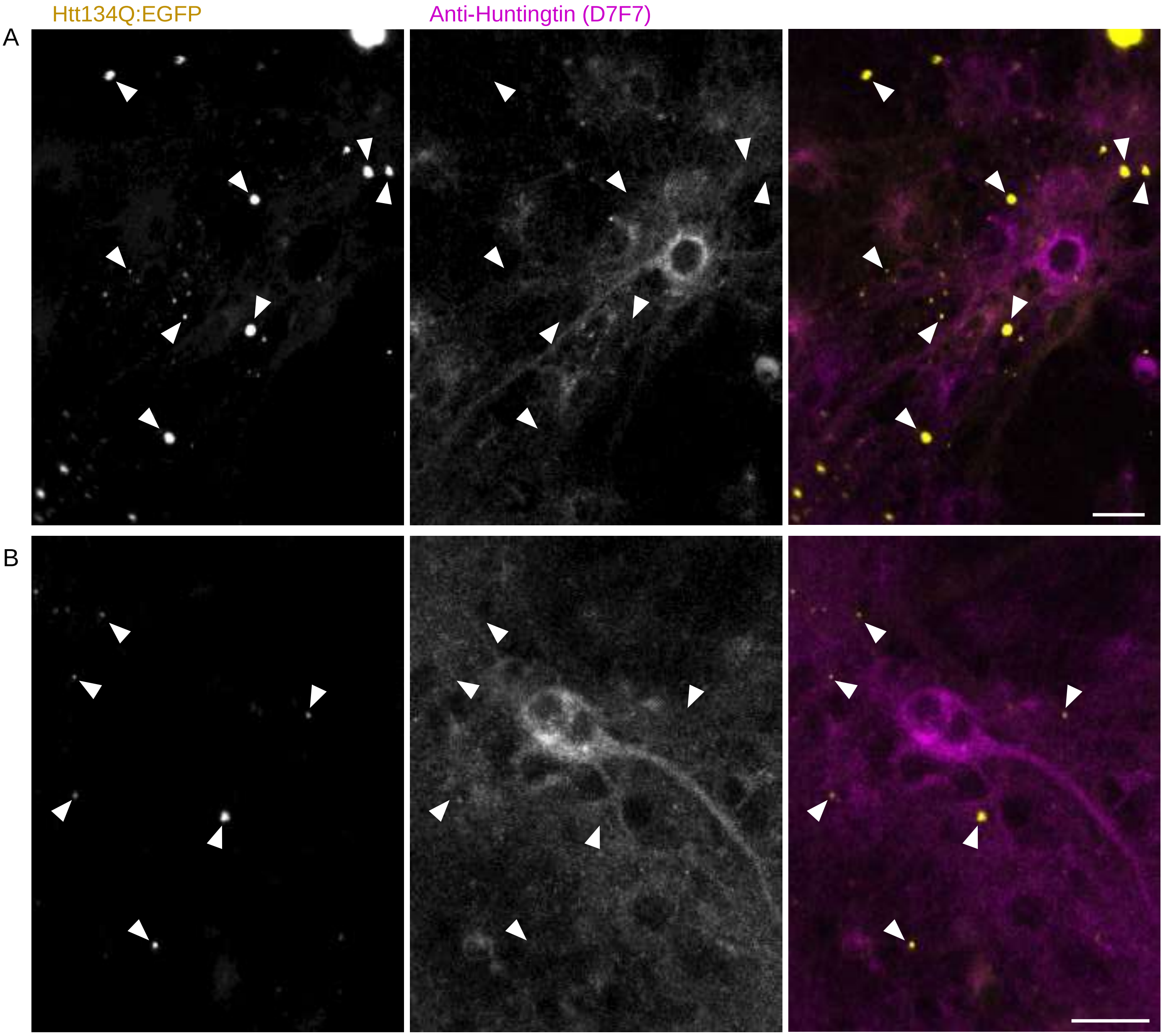

Supplementary Figure 6

Endogenous Htt does not colocalize with Htt134Q:EGFP aggregates. Cortical neurons plated on glass-bottom petri dishes expressing Htt134Q:EGFP were fixed and stained with an antibody against a synthetic peptide corresponding to residues surrounding Proline 1218 of human huntingtin. A, B) Two representative fields of view showing neurons expressing Htt134Q:EGFP and a number of Htt134Q:EGFP aggregates (arrowheads). No colocalization was found between Htt134Q:EGFP aggregates and endogenous Htt. 34 fields of view from three experiments from two independent cell culture preparations. Scale bars: 20µm.
