# Supplementary material for: Peripheral sequestration of huntingtin delays neuronal death and depends on N-terminal ubiquitination
Source: Commun Biol. 2024 Aug 18;7:1014. doi: 10.1038/s42003-024-06733-1 (PMC11330980; doi:10.1038/s42003-024-06733-1)
Supplement: Supplementary file 3 — Description of Additional Supplementary Materials [file 42003_2024_6733_MOESM3_ESM.pdf]

Description of Additional Supplementary Files

File name: Supplementary Data

Description: Underlying data of all graphs in manuscript.

File name: Supplementary Movie 1

Description: Time lapse of a neuron expressing Htt134Q:EGFP:T2A:mCherry. A neuron expressing Htt134Q:EGFP (green) and mCherry (red) followed for 22 days at 2 hour intervals. Note the disappearance of Htt134Q:EGFP cytosolic fluorescence and the appearance of numerous Htt134Q:EGFP puncta. A microglial cell is seen to traverse the field of view in the middle third of the video. Same neuron as that shown in Fig. 1.

File name: Supplementary Movie 2

Description: Htt134Q:EGFP aggregates appear to move along axons. A neuron expressing Htt134Q:EGFP (green) and mCherry (red) shown here for a 7 day segment (2 hour intervals). Note Htt134Q:EGFP puncta that move along what appear to be elongating axons
